# Effects of a complex mixture prepared from agrimonia, houttuynia, licorice, peony, and phellodendron on human skin cells

**DOI:** 10.1038/s41598-020-79301-2

**Published:** 2020-12-17

**Authors:** Kyung-Ha Lee, Jeong Pyo Lee, Wanil Kim

**Affiliations:** 1 Daegu Haany University, Gyeongsan, 38610 Republic of Korea; 2 Jincostech Corp., Siheung, 15083 Republic of Korea

**Keywords:** Biochemistry, Cell biology, Molecular biology, Physiology

## Abstract

Active ingredients derived from natural sources are widely utilized in many industries. Cosmetic active ingredients are largely derived from various plants. In this study, we examined whether a mixture of plant extracts obtained from agrimonia, houttuynia, licorice, peony, and phellodendron (hereafter AHLPP), which are well-known for their effects on skin, could affect skin barrier function, inflammation, and aging in human skin cells. We also determined whether AHLPP extracts sterilized using γ-irradiation (to avoid preservatives) retained their skin cell regulating activity. The AHLPP mixture could downregulate representative pro-inflammatory cytokines including IL 1-β and IL 7. Procollagen peptide synthesis was also increased by AHLPP treatment along with mRNA upregulation of barrier proteins such as filaggrin and desmoplakin. The AHLPP mixture showed an anti-aging effect by significantly upregulating telomerase activity in human keratinocytes. We further observed *TERT* upregulation and *CDKN1B* downregulation, implying a weakening of pro-aging signal transduction. Co-cultivation of a hydrogel polymer containing the AHLPP mixture with human skin cells showed an alteration in skin-significant genes such as *FLG*, which encodes filaggrin. Thus, the AHLPP mixture with or without γ-irradiation can be utilized for skin protection as it alters the expression of some significant genes in human skin cells.

## Introduction

Bioprospecting is a methodology that involves screening of bioactive molecules from various natural sources including fungi, algae, and bacteria. A large proportion of the biologically active ingredients used in cosmetology is derived from natural sources, which can be developed commercially with ease^1^. For example, astaxanthin from *Thraustochytrids*, *Rhotorula* spp., and *Phaffia rhodozyma* is commonly used as an antioxidant^2,3^. Hyaluronic acid purified from *Streptococcus thermophilus* is often used for anti-aging^4^. Bioprospecting is also common in screening medicines as most antibiotics in current clinical applications such as β-lactams, aminoglycosides, and tetracyclines are also derived from natural resources^5^. Therefore, many researchers in academia and the industry seek effective active ingredients from various natural sources.

In this study, we examined the skin-beneficial effects of a mixture of the extracts obtained from five herbal plants renowned for their skin benefits in the context of skin barrier function, anti-inflammation, and anti-aging. *Agrimonia pilosa* leaf extract has been shown to accelerate skin barrier restoration by activating Transient receptor potential vanilloid 3 (TRPV3)^6^. The antioxidant and anti-inflammatory effects of *A. pilosa* have also been suggested in multiple studies^7–10^. *Houttuynia cordata* is known to promote hair growth, upregulate filaggrin expression, and to ameliorate atopic dermatitis^11–13^. Licorice (*Glycyrrhiza uralensis*) is another herb well-known for its immune-modulating activity and anti-aging roles in UVA-induced photoaging^14–16^. *Paeonia lactiflora* is a species of herbaceous perennial plant with many varieties that show distinct flower types and colors, and provides substantial pharmacological benefits^17^. *Paeonia lactiflora* has been used for more than 1000 years in China to treat pain, inflammation, and immune disorders. Recent studies have shown that the *P. lactiflora* extract can alleviate allergic contact dermatitis, psoriasis, psoriatic arthritis, and skin damage caused by reactive oxygen species (ROS)^18–21^. *Phellodendron amurense* is commonly called the Amur cork tree and is known as one of the traditional herbal medicines. It is known to have the potential to alleviate skin pigmentation, inflammatory response, and acne vulgaris^22–24^. However, the combined effect of these five plant extracts on human skin cells is not known.

In the medical and cosmetic fields, hydrogels are used widely because of their excellent biocompatibility and multi-functionality. One of the benefits of using hydrogels is that they possess a stable three-dimensional structure that allows the storage of large amounts of decoctions containing various active ingredients^25–27^ Hydrogel polymers have different physiochemical properties according to the crosslinking methods used in their preparation, i.e., ion-crosslinking, chemical crosslinking, and radical crosslinking^28–32^. Among these, ion-crosslinking has several advantages such as reaction in mild conditions, easy manipulation, and less influence on active ingredients. Therefore, we considered that the ion-crosslinked hydrogel polymer may be suitable to deliver plant active extracts for skin applications.

Considering these points, we prepared a mixture of plant extracts from *Agrimonia*, *Houttuynia*, *Licorice*, *Paeonia*, and *Phellodendron* (described in the “Methods” section) and named it AHLPP; we then examined whether the mixture had a significant effect on the gene expression and cytokine profile of human epidermal keratinocytes and dermal fibroblasts. We further analyzed the effect of the AHLPP extract on some skin-significant genes with regard to skin-barrier function, inflammation, and aging. We also examined whether the AHLPP extract still functions after γ-irradiation to determine whether the mixture of extracts could be utilized without preservatives in further industrialization. In all the experiments we compared the activity of the control AHLPP extract and the irradiated-AHLPP extract. After concluding that the irradiated AHLPP extract was stable in terms of cellular activity in skin cells, we deposited the AHLPP extract into a hydrogel polymer and co-cultivated it with skin cells to determine whether a prototype of face sheet masks for commercial application could work in our in vitro system.

## Results

### The AHLPP mixture induced subtle cell death in human dermal fibroblasts and epidermal keratinocytes

We first extracted the water-soluble active ingredients from dried plants of agrimonia, houttuynia, licorice, peony, and phellodendron. Then, 65 g of *Phellodendron amurense Rupr*, 26 g of *Paeonia albiflora *Pallas, 14 g of *Agrimonia pilosa* Ledeb var*.* *japonica*, 5 g of *Glycyrrhiza uralensis *Fisch, and 3.5 g of *Houttuynia cordata *Thunb extracts were incubated with 1 L of water for 48 h, followed by lyophilization. We prepared the stock solutions of the lyophilized AHLPP extract in water for further analyses.

**Figure 1:**
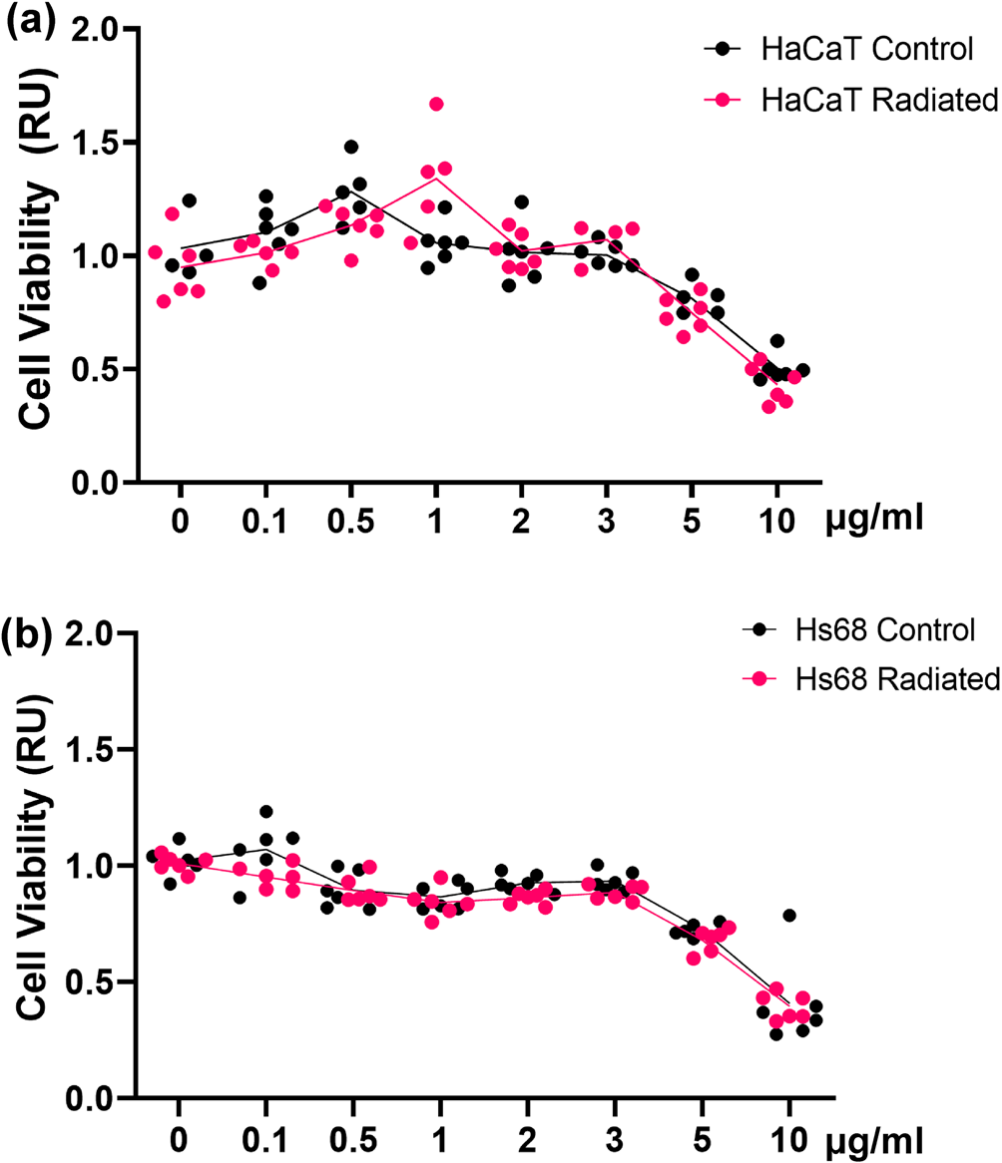
A mixture of plant extracts from agrimonia, houttuynia, licorice, peony, and phellodendron (hereafter AHLPP) induced subtle cell death in human epidermal keratinocyte and dermal fibroblasts. (**a**) HaCaT cells and (**b**) Hs68 cells were treated with the AHLPP extract for 48 h. Cell viability was assessed based on the activity of cytoplasmic dehydrogenases.

Cell viability was assessed by measuring the activity of cellular NAD/NADP dehydrogenases. Treatment of the Hs68 cells (human foreskin dermal fibroblasts) with AHLPP extract showed little cytotoxicity up to 3 μg/mL (Fig. 1a). We also examined the activity of the γ-irradiated AHLPP extract to determine whether we could utilize the extract without adding a preservative in further industrial processes. The result showed that pre-sterilization of the AHLPP extract by γ-irradiation did not change its effect on cell viability. We also determined the activity of the AHLPP extract on the aneuploid immortal human keratinocyte cell line, HaCaT, which showed low cytotoxicity similar to that in Hs68 cells (Fig. 1b). We thus concluded that AHLPP does not induce significant cytotoxicity under the concentrations of 5 µg/mL, indicating that controlled treatment with the extract might regulate skin cell physiology.

### The AHLPP mixture downregulates proinflammatory cytokines

**Figure 2:**
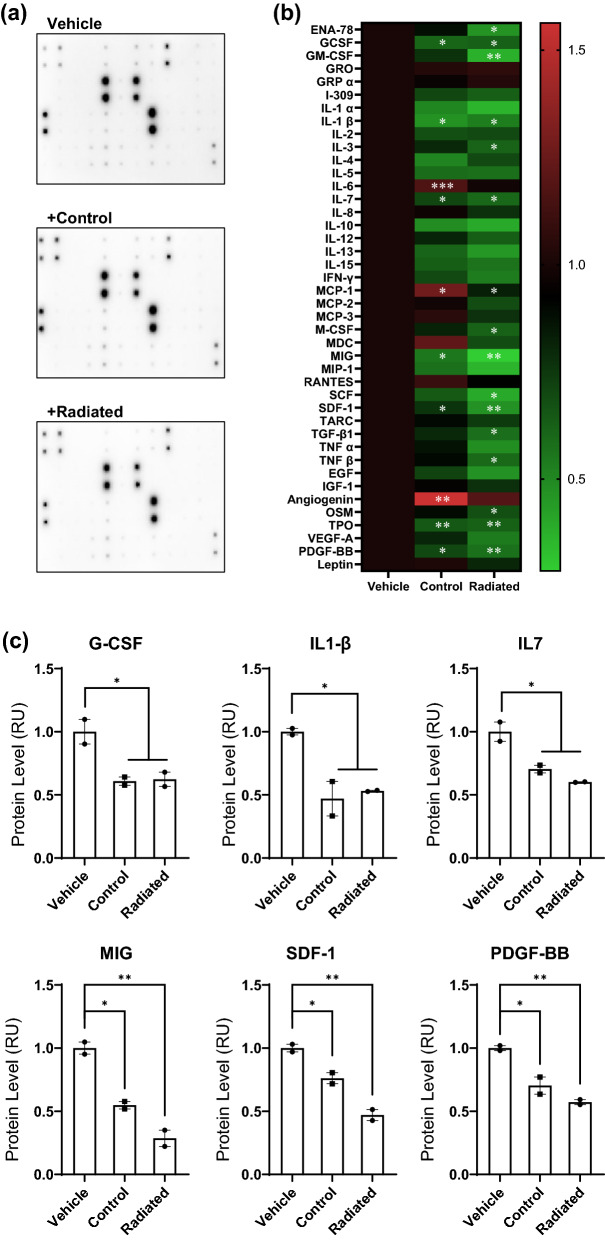
A mixture of plant extracts from agrimonia, houttuynia, licorice, peony, and phellodendron (hereafter AHLPP) downregulates proinflammatory cytokines in skin cells. (**a**) Representative immunoblot images of cytokine array analyses. After a 48 h incubation of HaCaT cells with 0.5 μg/mL of control and γ-irradiated AHLPP extracts, the supernatant from both treatments was harvested and analyzed. Densitometric analysis was performed using the Image J software. The same volume of water was used as vehicle control. (**b**) Heatmap of the cytokine array. Arbitrary value of the density was normalized to that of vehicle-treated supernatant. Red and green color indicate upregulation and downregulation of cytokines, respectively. Asterisks indicate statistical significance determined by one-way ANOVA. *p < 0.05, **p < 0.01, ***p < 0.005 (**c**) Protein expression of proinflammatory cytokines was analyzed. The same volume of water was used as vehicle control. Asterisks indicate statistical significance determined by one-way ANOVA. *p < 0.05, **p < 0.01.

After determining the treatable concentration of AHLPP for skin cells, we determined whether the AHLPP extract could regulate the secretion of inflammatory cytokines from epidermal keratinocytes (Fig. 2a). The epidermis is the foremost physical barrier to environmental hazards including microbes, allergens, pollutants, ionizing radiation, and repetitive contact^33^. Appropriate immune responses against the presence of microorganisms such as bacteria, fungi, and viruses on the skin play an important role in protecting the organism^34^. However, prolonged inflammatory responses harm an organism’s systemic health and often result in fatal autoimmune diseases or hyperinflammatory syndromes^35^. Sustained activation of inflammatory responses also triggers many unwanted side effects in aesthetic appreciation such as skin aging, wrinkle formation, and pigmentation^36–38^. Inflammation is also upregulated chronically in aged skin, which is often referred as inflamm-aging; thus, downregulation of inflammatory responses could be one of the strategies for anti-aging^39,40^.

We performed a heat map analysis of cytokine array results for some representative proinflammatory cytokines and found that granulocyte colony-stimulating factor (G-CSF), interleukin (IL) 1-β, IL 7, monokine induced by gamma interferon (MIG or CXCL9), stromal cell-derived factor 1 (SDF-1 or CXCL12), and platelet-derived growth factor subunit B (PDGF-BB) were significantly decreased by treatment with AHLPP extract as well as with γ-irradiated AHLPP extract (Fig. 2b). The CXCR2 ligands and G-CSF have been shown to induce severe intraepidermal neutrophilic inflammation and systemic neutrophilia via PKCα activation^41^. IL 1-β is one of the representative proinflammatory cytokines and IL 7 has also been shown to induce the secretion of inflammatory cytokines^42,43^. MIG is characteristic of certain skin inflammatory disorders such as contact hypersensitivity, interface dermatitis, lichenoid graft-versus-host disease (liGVHD), and lichen planus^44^. The SDF-1/CXCR4 axis has been shown to play important roles in skin inflammation and inflammatory angiogenesis^45^. The PDGF-BB/PDGFRβ activation is partly responsible for systemic sclerosis which is a chronic autoimmune disorder that can result in extensive skin damage^46^. Our results show that some representative proinflammatory cytokines were downregulated by the treatment of human keratinocytes with the AHLPP extract for 48 h (Fig. 2c).

Notably, secretion of proinflammatory IL 6, monocyte chemoattractant protein 1 (MCP1), and angiogenin^47–49^ was significantly increased by treatment with the AHLPP complex, but this secretion was not changed or increased with the γ-irradiated AHLPP complex. This result implies that γ-irradiation converted the bioactivity of the AHLPP complex such that the γ-irradiated complex could work in a somewhat different way compared to the control AHLPP complex. Overall, both the control and γ-irradiated AHLPP complex mixtures affected epidermal keratinocytes with a bias toward an anti-inflammatory fate.

### The AHLPP mixture enhances the skin barrier function

**Figure 3:**
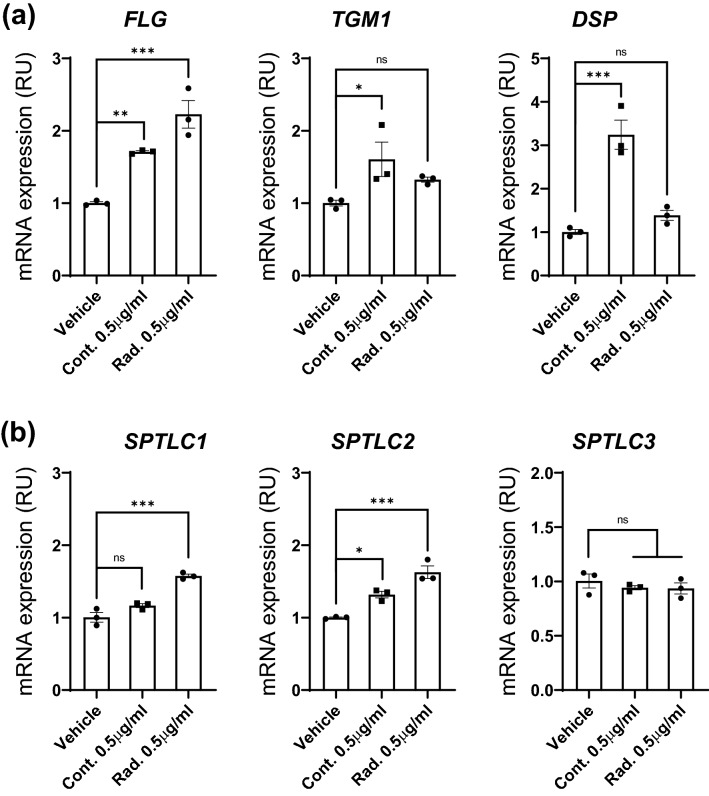
A mixture of plant extracts from agrimonia, houttuynia, licorice, peony, and phellodendron (hereafter AHLPP) enhances skin barrier function. HaCaT cells were treated with 0.5 μg/mL of control and γ-irradiated AHLPP extracts for 48 h. Quantitative real-time PCR analyses were performed to assess mRNA expression of the indicated genes. *G6PD* was used as a loading control. (**a**) Analyses of genes involved in skin barrier function: Filaggrin (*FLG*), Transglutaminase 1 (*TGM1*), Desmoplakin (*DSP)*. (**b**) Analyses of genes involved in skin ceramide synthesis: Serine Palmitoyltransferase Long Chain Base Subunit (*SPTLC*s). The same volume of water was used as vehicle control. Asterisks indicate statistical significance determined by one-way ANOVA. *p < 0.05, **p < 0.01, ***p < 0.005, *ns* non-significant.

We next examined the skin barrier function because proper protection from harmful microbes and environmental pollutants such as UV rays or ambient fine particles is essential for the skin as well as the systemic health of the organism^50^. The mRNA expression of *FLG*, which encodes filaggrin that plays an important role in the formation of the keratin network in corneocytes at the stratum corneum^51^, was first determined to check whether AHLPP could affect the skin barrier function (Fig. 3a). Treatment with both control and γ-irradiated AHLPP significantly upregulated the expression of filaggrin. We also examined other barrier function genes including *TGM1* and *DSP*. *TGM1* encodes transglutaminase 1 enzyme, which is expressed in the upper spinous and granular layers beneath the outermost horny layer^52^. The roles of transglutaminase 1 encompass cross-linking of structural skin proteins such as involucrin, loricrin, and trichohyalin^53^. *DSP* encodes desmoplakin protein, which serves as one of the major components for epidermal integrity by building desmosomes between keratinocytes^54^. Notably, the mRNA expression of transglutaminase 1 and desmoplakin was significantly increased only with the non-irradiated AHLPP extract. We thus assume that γ-irradiation sterilized the AHLPP extract as well as altered the physical or chemical composition of the compound. As observed from the data in Fig. 2, some cytokines are differentially expressed in the AHLPP-treated cells. One of the reasons for this is the structural change that arises from γ-irradiation and converts some active ingredients in the AHLPP extract to inactive forms, which cannot stimulate human skin cells. Thus, γ-irradiation could be utilized for manipulating biological activity as well as for sterilizing the ingredient.

Next, we examined whether treatment with the AHLPP extract could influence the synthesis of ceramide, which is one of the major lipids between the outermost corneocytes and helps in barrier function (Fig. 3b). Serine Palmitoyltransferase Long Chain Base Subunit (*SPTLC*) genes encode serine palmitoyl transferases that are essential for the de novo synthesis of 3-ketosphonganine, which works as a precursor of various skin ceramides^55,56^. The SPTLC1 and SPTLC2 proteins function as a rate-limiting step in ceramide and sphingolipid metabolism^56^. *SPTLC3* is an isoform of the *SPTLC2* gene and participates in the generation of short-chain sphingoid bases^57^. Treatment with the γ-irradiated AHLPP extract significantly upregulated the mRNA expression of *SPTLC1* and *SPTLC2*. However, the non-irradiated AHLPP extract only changed the mRNA expression of *SPTLC2*. Taken together, we suggest that treatment with the AHLPP extract could regulate ceramide synthesis in the epidermis by increasing the mRNA amounts of SPTLC1 and SPTLC2, which are essential enzymes that are required at the initial stage of biosynthesis for cutaneous sphingoid bases.

### The AHLPP mixture promotes collagen synthesis in Hs68 cells

**Figure 4:**
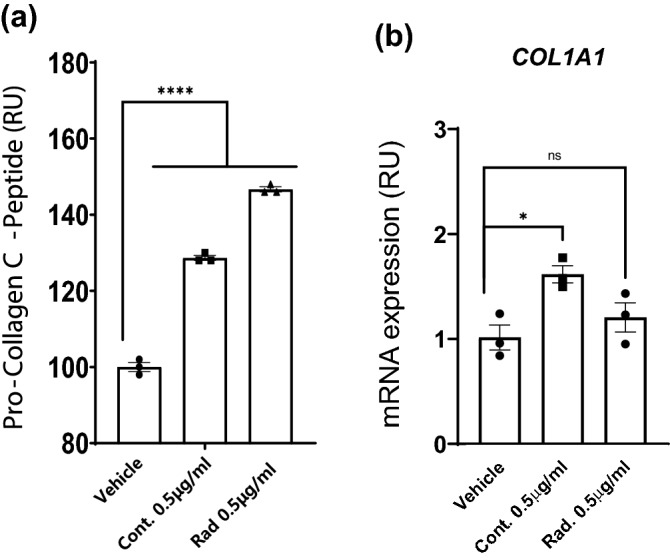
A mixture of plant extracts from agrimonia, houttuynia, licorice, peony, and phellodendron (hereafter AHLPP) promotes collagen synthesis in Hs68 cells. Hs68 cells were treated with indicated concentration of control and γ-irradiated AHLPP extracts. (**a**) Protein levels of procollagen C-terminal peptides in supernatant were determined via antibody-based colorimetric analysis. (**b**) mRNA expression of Collagen type 1 alpha 1 (*COL1A1*) was analyzed. The same volume of water was used as vehicle control. Asterisks indicate statistical significance. One-way ANOVA revealed statistical significance. *p < 0.05, ****p < 0.0001, *ns* non-significant.

We next considered whether the AHLPP extract could impact the dermal collagen network dynamic. Collagen plays functional and structural roles in the dermis and is progressively weakened by aging or external stresses such as UV rays and chemicals^58^. Collagen is first synthesized as pre-procollagen, which is further processed to procollagen triple-helix in the endoplasmic reticulum. Procollagen is next secreted into the extracellular space and further releases cleaved pro-peptide to form mature collagen fibrils via crosslinking to form a collagen fiber^59^. We assessed the protein amount of cleaved procollagen C-terminal peptide to determine whether the AHLPP extract can regulate collagen synthesis in skin cells (Fig. 4a). The supernatant of Hs68 human fibroblast cells treated with AHLPP extracts showed elevated amounts of procollagen C-peptides. We also determined the mRNA expression of *COL1A1*, which is one of the most abundant types of skin collagen (Fig. 4b). Notably, *COL1A1* expression was significantly upregulated only in Hs68 cells treated with 0.5 μg/mL of the non-irradiated extract. This implies that treatment with the non-irradiated AHLPP extract facilitated procollagen processing rather than increasing the gene transcription. Taken together, these results suggest that AHLPP treatment of human dermal fibroblasts could increase collagen protein synthesis and that the extract could be utilized as an active cosmetic ingredient.

### Anti-aging effect of the AHLPP mixture

**Figure 5:**
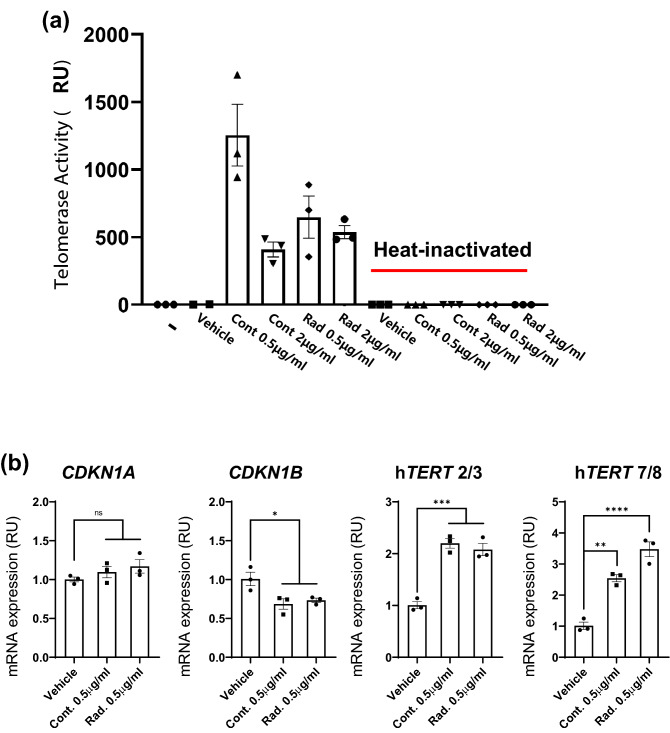
Anti-aging effect of a mixture of plant extracts from agrimonia, houttuynia, licorice, peony, and phellodendron (hereafter AHLPP). HaCaT cells were treated with indicated concentration of control and γ-irradiated AHLPP extracts. (**a**) Treated cells were harvested and disrupted in non-denatured buffer. Activity of telomerase was determined based on relative amplification of external telomerase substrates (TS primer). Heat-inactivated cell lysate was used as a negative control. (**b**) Analyses of aging-associated genes: Cyclin Dependent Kinase Inhibitor 1A (*CDKN1A*: p21), Cyclin Dependent Kinase Inhibitor 1A (*CDKN1B*: p16), Telomerase reverse transcriptase (*TERT*). The same volume of water was used as vehicle control. Asterisks indicate statistical significance. One-way ANOVA revealed statistical significance. *p < 0.05, **p < 0.01, ***p < 0.005, ****p < 0.0001, *ns* non-significant.

Human epidermal keratinocytes actively proliferate and differentiate to maintain the integrity of skin. However, keratinocytes have a finite lifespan and aged cells show reduced cell division and senescence phenotypes^60^. This sometimes makes it difficult for aged people to replenish the damaged regions of their skin^61^. One of the major players in the regulation of keratinocyte aging is telomerase, which adds protective telomeric repetitive sequences at the end of chromosomes^62,63^. We thus analyzed whether the AHLPP extract could regulate human telomerase enzyme activity in HaCaT cells (Fig. 5a). Treatment with control as well as γ-irradiated AHLPP extracts significantly upregulated human telomerase activity in HaCaT cells. We also heat-inactivated the lysates to demonstrate that the results were derived from active enzymes in live cells. This implies that AHLPP extracts might directly affect the enzymatic activity of telomerase to help maintain a pool of viable keratinocytes by preventing premature aging.

We next examined whether treatment with these extracts could regulate the expression of senescence-associated genes such as *CDKN1A* and *CDKN1B*. *CDKN1A* encodes the p21 protein that inhibits the CDK2/Cyclin E complex to induce cell cycle arrest^64^. The p21 protein is also induced by telomere shortening via the p53 pathway in cooperation with p16. Thus, we examined whether p21 and p16 expression was affected by treatment with the AHLPP extracts (Fig. 5b). Treatment of HaCaT cells with AHLPP extracts for 48 h significantly downregulated the mRNA expression of *CDKN1B* which encodes p16. However, p21 mRNA expression showed no change. We also analyzed the mRNA expression of human *TERT* by amplifying the splice junctions at exon 2/3 and exon 7/8 in order to assess the total transcripts and catalytic active forms of *TERT*, respectively^65^. The results show that treatment with both control and γ-irradiated AHLPP extracts triggered significant upregulation of both total and catalytic active forms of human TERT. Taken together, these results suggest that the AHLPP extract could be used as an anti-aging ingredient to regulate the activity of human telomerase enzymes and the mRNA expression of aging-associated genes such as *CDKN1A*, *CDKN1B*, and *TERT*.

### Analysis of the γ-irradiated AHLPP extract incorporated in a hydrogel polymer

We further deposited the γ-irradiated AHLPP extract in a hydrogel polymer (1% w/w) without any preservatives to determine the combined cytotoxicity and effect of the γ-irradiated extract. Cultivation of γ-irradiated hydrogel polymer filled with control and γ-irradiated extracts on agar plates specific to *Pseudomonas aeruginosa* (ATCC No. 9027), *Escherichia coli* (ATCC No. 8739), *Candida albicans* (ATCC No. 10231), and *Acinetobacter baumannii* (ATCC No. 16404) revealed no microbial contamination upon culture for up to 14 days (Table 1). We assume that the extract is sterile when prepared but can be easily contaminated outside its packaging. Thus, the hydrogel polymer and the AHLPP extract without preservatives should be used in the production of disposable patches or sheet face masks that must be consumed within 30 min of unpacking.

**Table 1:** Microbial growth assessment of the γ-irradiated hydrogel polymer filled with the control vs. γ-irradiated mixture of plant extracts from agrimonia, houttuynia, licorice, peony, and phellodendron (AHLPP) extracts.

	*S. aureus*	*P. aeruginosa*	*E. coli*	*C. albicans*	*A. baumannii*
Control extract (7 days)	Not detected	Not detected	Not detected	Not detected	Not detected
Control extract (14 days)	Not detected	Not detected	Not detected	Not detected	Not detected
γ-Irradiated extract (7 days)	Not detected	Not detected	Not detected	Not detected	Not detected
γ-Irradiated extract (14 days)	Not detected	Not detected	Not detected	Not detected	Not detected

**Figure 6:**
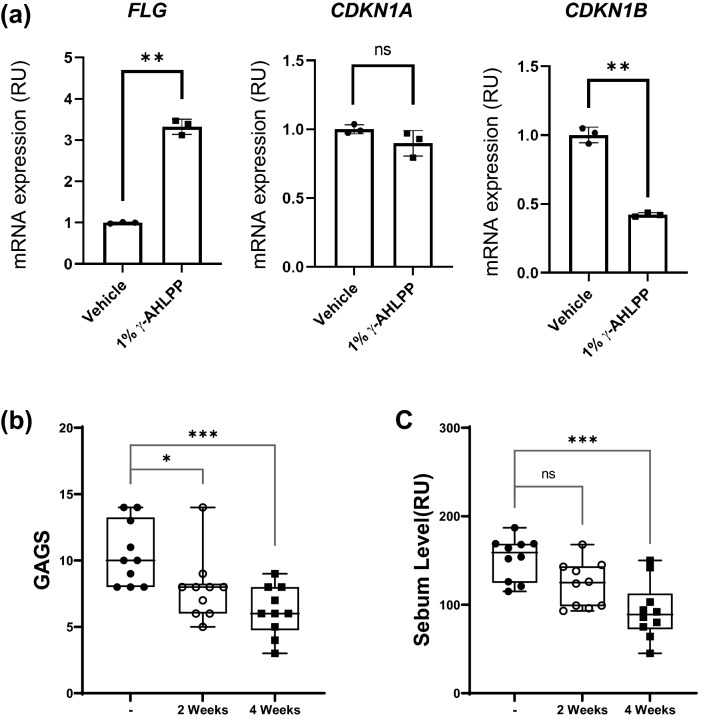
A mixture of plant extracts from agrimonia, houttuynia, licorice, peony, and phellodendron (hereafter AHLPP). (**a**) Deposition of γ-irradiated AHLPP extract in hydrogel-polymer-regulated mRNA expression of co-cultivated cells. One percent of γ-irradiated AHLPP extract was deposited in hydrogel by volume (vol %) and incubated with HaCaT cells for 48 h. Harvested cells were analyzed for mRNA quantitation. The same volume of water was used as vehicle control. Asterisks indicate statistical significance. T-test revealed statistical significance. **p < 0.01, *ns* non-significant. (**b**) Global Acne Grading System (GAGS) was determined after treatment of the AHLPP extract with hydrogel polymer for 2 and 4 weeks on human inflamed acne skin. (**c**) Sebum level of human skin was assessed after treatment of the AHLPP extract with hydrogel polymer for 2 and 4 weeks. Ten individuals participated in the clinical analysis. One-way ANOVA revealed statistical significance. *p < 0.05, **p < 0.01, ***p < 0.005, *ns* non-significant.

We next cultivated the γ-irradiated hydrogel polymer with HaCaT cells for 48 h and analyzed the mRNA expression of *FLG* to examine whether the extracts released from the hydrogel polymer could regulate the gene expression of co-cultivated cells (Fig. 6). The treated cells showed no significant cell death and an increased mRNA expression of *FLG* implying that the hydrogel-deposited AHLPP extract could be utilized for skin benefit. We further examined the mRNA expression of *CDKN1A* and *CDKN2A* to assess whether the extract could regulate cellular senescence-associated genes. *CDKN2A* was significantly downregulated in accordance with our analysis as shown in Fig. 5. These results indicate that the γ-irradiated AHLPP extract retains its biological activity even after being deposited in the hydrogel polymer to regulate the mRNA expression of adjacent cells.

We next determined whether the application of the hydrogel polymer with the AHLPP extract could treat acne-prone skin. This clinical study was conducted by recruiting 10 male and female adults with acne aged between 20 and 35 years old. In this test, the visual evaluation according to the Global Acne Grading System (GAGS) and the sebum improvement evaluation using a Sebumeter (SKIN-OMAT, Cosmomed GmbH, Germany) were performed together to evaluate the efficacy of the AHLPP extract in hydrogel polymer on acne-prone skin^66^. The result indicates that the use of AHLPP showed an improvement after more than 2 weeks of use (Fig. 6b). In addition, the decrease of sebum production was statistically significant (*p* < 0.001), showing nearly 40% improvement after 4 weeks of use. These results imply that the product can be considered for skin benefit.

## Discussion

It is claimed that many natural compounds demonstrate physiological activity toward human skin cells to regulate barrier function, inflammation, and aging. Among these, we selected agrimonia, houttuynia, licorice, peony, and phellodendron plants to determine whether a combination of their extracts could be used for skin benefit. We also prepared γ-irradiated extracts to avoid the use of non-preferential conservatives for sterilization and examined whether their activity is still maintained after radiation exposure. We further checked whether γ-irradiation of the AHLPP extract could modulate its bioactivity.

Radiosterilization was first performed in 1895 and its use in industrial fields has increased significantly every year^67^. γ-Irradiation is one of the most popular methods in radiation sterilization and sterilization of biomolecules using γ-rays has many advantages over heat or chemical sterilization^68^. These include minimal generation of heat, ease in dosage control, independence from residual chemicals, and application in terminal processing^67,68^. Thus, we considered that this method could be used to sterilize the extract as well as the hydrogel polymer for improved cosmetic application.

Figure 2 shows upregulation of anti-inflammatory cytokines and downregulation of proinflammatory cytokines. Secretion of some inflammatory cytokines such as granulocyte–macrophage colony-stimulating factor (GM-CSF), IL 3, macrophage colony-stimulating factor (M-CSF), stem cell factor (SCF), transforming growth factor-beta 1 (TGF-β1), tumor necrosis factor-beta (TNF β), and oncostatin M (OSM) was not altered by treatment with the AHLPP extract, whereas treatment with γ-irradiated AHLPP extract showed significant changes in their expression (Fig. 2b). Although we did not determine the reason for this phenomenon, it is well-known that γ-irradiation induces changes in the physical and chemical properties of natural compounds^69,70^. Thus, we assumed that γ-irradiation sterilized the AHLPP extracts as well as enhanced their activity in some cases. Further studies are needed to determine the molecular changes induced by γ-irradiation for safe and efficient utilization of the AHLPP extract.

Synergism between plant extracts has been considered in the development of many pharmaceutical products such as diabetes mellitus and infectious diseases^71–73^. A recent report also suggested that a combination of the extracts of *Cornus officinalis, Rosa multiflora, Lespedeza bicolor*, *Platycladus orientalis*, and *Castanea crenata* showed preventive effects on atopic dermatitis-like skin lesions^74^. Plant extracts have been regarded as a source of the bioactive substances in plants for a long time, and many studies have been already conducted on them^75–77^. In this study, we analyzed the potential physiological activity of the AHLPP extract as active material in cosmetics or pharmaceuticals.

Unfortunately, we could not identify a single active component in the AHLPP mixture. This could be one of the major limitations of the present study. However, many studies reported putative active components in the AHLPP. Bioactive compounds of the *H. cordata* extract include flavonoids and polyphenols such as quercetin, rutin, hyperin, protocatechuic acid, chlorogenic acid, and vanillic acid^78^. Phytochemical investigation of *A. pilosa* showed that flavonoids, triperterpenes, isocoumarin, and phenolic acids such as tiliroside, quercetin, luteolin, and apigenin were the main components of its extract^79^. *Phellodendron amurense* has been shown to contain myrcene, β-elemol, and amurensin^80,81^. Paeonilactone-C and benzoylpaeoniflorin were the major active ingredients of *P. lactiflora*^82^. The key active compounds of *G. uralensis* encompass glycyuralin D, isoangustone A, 7-*O*-methylluteone, and glyasperin D^83^.

Thus, we prepared the AHLPP extract from herbal plants which are known to exert skin-beneficial functions. We examined whether the extract could regulate inflammation, skin barrier function, and aging in HaCaT human keratinocyte cells. We also subjected the AHLPP extract to γ-irradiation to determine whether we could use the extract without any preservatives. We found that the γ-irradiated AHLPP extracts retained biological activity toward human skin cells, and showed an enhanced effect on the regulation of the mRNA and protein expression of some genes. We also showed that the γ-irradiated AHLPP extract deposited in a hydrogel polymer also regulated gene expression. Thus, we suggest that AHLPP extract can be used as an active cosmeceutical ingredient for the treatment of human skin and that its preparation using γ-irradiation might provide the extract with additional benefits.

## Methods

### Preparation of AHLPP extracts

The extracts were decoctions prepared by boiling 500 g each of *H. cordata*, *A. pilosa*, *P. amurense* bark, *P. lactiflora* root, and *G. uralensis* (Licorice) root with a ten-fold volume of water for 3 h, respectively. After three rounds of filtration through a 0.45-μm pore membrane, the extracts were concentrated using a rotary evaporator (HS-2000NS, Hanshin Scientific, Seoul, South Korea). Each extract was further freeze-dried for preparing the complex (FD-5518, Ilshin Lab Co., Ltd., Seoul, South Korea). The AHLPP extract complex was prepared by incubating *P. amurense *Rupr extract (65 g), *P. albiflora *Pallas extract (26 g), *A.* *pilosa* Ledeb var*.* *japonica* extract (14 g), *G. uralensis *Fisch extract (5 g), and *H. cordata *Thunb extract (3.5 g) for 48 h in 1 L of water, followed by lyophilization. The ratio was determined experimentally.

### Gamma irradiation of AHLPP extracts

The AHLPP extract was exposed to 3 kGy of γ-irradiation from a high-power MB10-30 accelerator used for research and in industrial use at 10 meV 30 kW (Mevex, Stittsville, Canada).

### Preparation of hydrogel polymer containing the AHLPP extract

Carrageenan, gellan gum, carob gum (CP Kelco, Atlanta, GA, USA), glycerin (Palm-Oleo Sdn. Bhd., Selangor, Malaysia), ethylenediaminetetraacetic acid (AkzoNobel, Amsterdam, Netherlands), polyglyceryl laurate (BSKorea, Seoul, South Korea), and lavender oil (Bontoux, Saint-Auban-sur-l’Ouvèze, France) were homogenized for fabricating a hydrogel polymer (HM1QT, K&S company, Seoul, South Korea). Briefly, 1% of carrageenan, gellan gum, and carob gum were mixed with 15% of glycerin solution on a stirrer at room temperature. Next, 0.03% of EDTA was added to the hydrogel solution and mixed for 5 min at 2500 rpm at 75 °C. Then, 0.01% lavender oil and 0.1% polyglyceryl laurate solution were prepared and mixed with the hydrogel solution for 5 min at 2500 rpm. Finally, 1% AHLPP extract was added to the hydrogel solution and mixed at 2500 rpm at 75 ℃ for 5 min. The contents of the hydrogel are described in Table 2.

**Table 2:** Composition of the hydrogel polymer with the mixture of plant extracts from agrimonia, houttuynia, licorice, peony, and phellodendron (AHLPP) extract.

Contents	Percentage (%, w/w)
Distilled water	To 100
Ethylenediaminetetraacetic acid	0.03
Glycerin	15.00
Carrageenan	1.00
Gellan gum	1.00
Carob gum	1.00
Polyglyceryl laurate	0.10
Lavender Oil	0.01
AHLPP extract	1.00

### Cell culture

Human dermal fibroblast, Hs68, and human epidermal keratinocyte, HaCaT, cell lines were cultured in Dulbecco’s modified Eagle medium (DMEM) supplemented with 10% fetal bovine serum (FBS) and 1% penicillin/streptomycin. Cells were maintained under 5% CO_2_ at 37 °C. The number of viable cells was determined using the CCK-8 assay kit (ApexBio Technology, Houston, TX, USA) following the manufacturer’s instruction.

### Cytokine analysis

The HaCaT cells were treated with AHLPP or γ-irradiated-AHLPP extracts for 48 h. After clarification of the supernatant at 15,000×*g* for 30 min, a cytokine array was performed following the manufacturer’s instruction (AAH-CYT-3, RayBiotech Life, Peachtree Corners, GA, USA). Arbitrary protein amounts were quantitated using the Image J software (NIH, Bethesda, MD, USA) and visualized using Prism 8 (GraphPad Software, San Diego, CA, USA). Statistical analyses were performed with Prism 8 based on one-way ANOVA.

### Quantitative real-time PCR analysis

Total RNA was extracted from the cultivated cells using TRIzol (Thermo Fisher Scientific, Waltham, MA, USA) reagent according to the manufacturer’s instruction. Next, 100 ng of RNA was reverse transcribed using the iScript cDNA kit (Bio-Rad, Hercules, CA, USA) followed by gene expression analysis using TaqMan Universal PCR Master Mix (Thermo Fisher Scientific, Waltham, MA, USA) on the QuantStudio 3 Real-Time PCR System (Thermo Fisher Scientific, Waltham, MA, USA). Probes for each gene were selected from the Universal Probe Library (Roche, Basel, Switzerland) using the ProbeFinder Assay Design Software. The primer sequences and probe numbers for each gene are described below.

- FLG-GGACTCTGAGAGGCGATCTG/TGCTCCCGAGAAGATCCAT (#38)
- TGM1-ACATCCCTTACCATGGACATCT/GTCGTTCCACACATGGAAGTT (#1)
- DSP-GGAAATTGAGAAATTCCAAAAGC/CTGCAGCCTTGCCTTGTC (#1)
- SPTLC1-TTGTCCTCTTCCAGAATTGGTT/GCTCTCCTAGGACTCCAAATGA (#65)
- SPTLC2-CCTCTTTCAGCAGATCACATCA/GGGCTTTTGACATCTCCTAGC (#72)
- SPTLC3-GTTTTGGAGCTTCAGGAGGTT/CCCGTAAATAATCCACGAGGT (#11)
- COL1A1-GGGATTCCCTGGACCTAAAG/GGAACACCTCGCTCTCCA (#67)
- TERT 2/3-AAGCATGCCAAGCTCTCG/CAGGATCTCCTCACGCAGAC (#17)
- TERT 7/8-GCGTAGGAAGACGTCGAAGA/ACAGTTCGTGGCTCACCTG (#52)
- CDKN1A-CCAGCACTCCCTCTAAGCAG/GCTCCGACATGTGGTCCT (#19)
- CDKN2A-CACATTCATGTGGGCATTTC/TGCTTGTCATGAAGTCGACAG (#75)
- G6PD-AACAGAGTGAGCCCTTCTTCA/GGAGGCTGCATCATCGTACT (#5)

### Procollagen determination

Procollagen C-peptide was measured using a Procollagen Type I C-Peptide (PIP) EIA Kit following the manufacturer’s instruction (Takara Bio Inc., Shiga, Japan). Briefly, Hs68 cells were treated with the AHLPP extract for 48 h at the indicated concentration. After clarification, the supernatant was harvested and added to an anti-PIP monoclonal antibody-coated plate and incubated for 3 h at 37 °C. Peroxidase-labeled secondary antibody was then applied, and the results were visualized by measuring the optical density at 450 nm.

### Real-time quantitative telomeric repeats amplification protocol (RQ-TRAP) analysis

Measurement of telomerase activity by quantitative real-time PCR was performed as described previously^84^. Briefly, PBS-washed cells were lysed with NP-40 buffer (10 mM Tris–HCl, pH 8.0, 1 mM magnesium chloride, 1 mM EDTA, 1% NP-40, 0.25 mM sodium deoxycholate, 10% glycerol, 150 mM sodium chloride, 5 mM 2-mercaptoethanol, 0,1 mM AEBSF or 4-(2-Aminoethyl)benzenesulfonyl fluoride hydrochloride) and incubated with telomerase primer TS and anchored return primer ACX in SYBR Green qPCR Master Mix (Thermo Fisher Scientific, Waltham, MA, USA) for 40 min at 25 °C. Telomerase was deactivated for 5 min at 95 °C, followed by amplification for 30 cycles of 95 °C for 30 s and 60 °C for 90 s. Heat-inactivated controls were assayed on every plate.

### Approval for human experiments

All clinical tests in this study were performed and approved by the Korea Institute of Dermatological Sciences (Cheongju, South Korea). All experiments were performed in accordance with relevant guidelines and regulations from the Korean Ministry of Health and Welfare. Informed consent was obtained from all participants. The internal case number for the tests is KIDS-AIB030-PTP.

## Data Availability

All relevant data are included in the manuscript and its associated files.
